# Caffeine-Induced Activated Glucocorticoid Metabolism in the Hippocampus Causes Hypothalamic-Pituitary-Adrenal Axis Inhibition in Fetal Rats

**DOI:** 10.1371/journal.pone.0044497

**Published:** 2012-09-06

**Authors:** Dan Xu, Benjian Zhang, Gai Liang, Jie Ping, Hao Kou, Xiaojun Li, Jie Xiong, Dongcai Hu, Liaobin Chen, Jacques Magdalou, Hui Wang

**Affiliations:** 1 Department of Pharmacology, Basic Medical School of Wuhan University, Wuhan, China; 2 Research Center of Food and Drug Evaluation, Wuhan University, Wuhan, China; 3 Department of Orthopedic Surgery, Zhongnan Hospital of Wuhan University, Wuhan, China; 4 UMR 7561 CNRS-Nancy Université, Faculté de Médicine, Vandoeuvre-lès-Nancy, France; Hôpital Robert Debré, France

## Abstract

Epidemiological investigations have shown that fetuses with intrauterine growth retardation (IUGR) are susceptible to adult metabolic syndrome. Clinical investigations and experiments have demonstrated that caffeine is a definite inducer of IUGR, as children who ingest caffeine-containing food or drinks are highly susceptible to adult obesity and hypertension. Our goals for this study were to investigate the effect of prenatal caffeine ingestion on the functional development of the fetal hippocampus and the hypothalamic-pituitary-adrenal (HPA) axis and to clarify an intrauterine HPA axis-associated neuroendocrine alteration induced by caffeine. Pregnant Wistar rats were intragastrically administered 20, 60, and 180 mg/kg·d caffeine from gestational days 11–20. The results show that prenatal caffeine ingestion significantly decreased the expression of fetal hypothalamus corticotrophin-releasing hormone. The fetal adrenal cortex changed into slight and the expression of fetal adrenal steroid acute regulatory protein (StAR) and cholesterol side-chain cleavage enzyme (P450scc), as well as the level of fetal adrenal endogenous corticosterone (CORT), were all significantly decreased after caffeine treatment. Moreover, caffeine ingestion significantly increased the levels of maternal and fetal blood CORT and decreased the expression of placental 11β-hydroxysteroid dehydrogenase-2 (11β-HSD-2). Additionally, both *in vivo* and *in vitro* studies show that caffeine can downregulate the expression of fetal hippocampal 11β-HSD-2, promote the expression of 11β-hydroxysteroid dehydrogenase 1 and glucocorticoid receptor (GR), and enhance DNA methylation within the hippocampal 11β-HSD-2 promoter. These results suggest that prenatal caffeine ingestion inhibits the development of the fetal HPA axis, which may be associated with the fetal overexposure to maternal glucocorticoid and activated glucocorticoid metabolism in the fetal hippocampus. These results will be beneficial in elucidating the developmental toxicity of caffeine and in exploring the fetal origin of adult HPA axis dysfunction and metabolic syndrome susceptibility for offspring with IUGR induced by caffeine.

## Introduction

Intrauterine growth retardation (IUGR) refers to the poor growth of a baby while in the mother's womb during pregnancy. Specifically, it is defined as a developing baby weighing 10% or two standard deviations less than the mean body weight of other babies at the same gestational age [1]. IUGR is often considered a root cause of a series of perinatal diseases, including fetal distress, neonatal asphyxia and perinatal death. In addition, the adverse effects of IUGR can be prolonged into maturity and result in physical and mental stunting, as well as increased susceptibility to adult metabolic syndromes [2]. Epidemiological investigation [3] has revealed that the incidence of adult metabolic syndrome in IUGR-affected infants is 2.53-fold higher than that of normal fetuses. As a high-risk group susceptible to adult metabolic syndrome, this baby group has already drawn considerable attention.

The etiology of IUGR is largely attributed to an adverse exogenous environment during pregnancy. Caffeine is a xanthine alkaloid widely consumed in the form of coffee, tea, soft beverages, food and some analgesic drugs. For example, 52% of all persons in the US over 10 years of age consume coffee [4], and the consumption of caffeinated beverages during pregnancy is quite common [5]. Clinical investigations and experiments have demonstrated that caffeine ingestion during pregnancy results in reproductive and developmental toxicities [6], [7]. In our previous study, we demonstrated that caffeine ingestion before and during pregnancy increases the incidence of adsorption and stillbirth in mice, and the live fetal growth and developmental status as estimated from several indices (such as body weight and body length) is severely retarded [8]. Reports also indicate that children who ingest caffeine-containing food or drinks are highly susceptible to metabolic syndromes such as obesity and hypertension [9], [10]. These results suggest that IUGR induced by prenatal caffeine ingestion may increase the susceptibility to adult metabolic syndrome. The underlying mechanism in reference to the fetal origin, however, has not been clarified.

The well accepted hypothesis pertaining to the underlying mechanism of IUGR, as well as the high susceptibility to adult metabolic syndrome, is “intrauterine endocrine metabolic programming” proposed by Fowden [11], which states that adverse intrauterine environment (i.e., stress, hypoxemia and malnutrition) changes the development of the fetal hypothalamic-pituitary-adrenal (HPA) axis, retarding fetal growth and increasing the sensitivity to metabolic hormones in peripheral tissues. After birth, these babies would present an increased HPA axis sensitivity, which would accelerate the process of metabolic disturbance. Recent reports also have emphasized that the altered programming of the HPA axis is most likely involved in the underlying mechanism behind the intrauterine origin of adult metabolic syndrome [12], [13]. Glucocorticoid (GC) is known to be an important factor in insulin resistance and metabolic syndrome in adults [14], [15]. Circulatory GC has been reported to regulate a series of signaling pathways related to insulin resistance, including the insulin-like growth factor/insulin [16], [17], adiponectin [18], [19] and leptin [20] signaling pathways.

Our recent study and research data from other laboratories have reported that caffeine elevates GC levels in both humans and animals [21]–[24]. Our other studies have confirmed that prenatal nicotine and ethanol exposure increased the expression of adrenal steroid acute regulatory protein (StAR) and cholesterol side-chain cleavage enzyme (P450scc) in pregnant rats but decreased their expression in IUGR fetuses. Additionally, nicotine and ethanol reduced the expression of placental 11β-hydroxysteroid dehydrogenase-2 (11β-HSD-2), which is responsible for the inactivation of maternal-derived glucocorticoid (GC) [25], [26]. These observations suggest that prenatal nicotine and ethanol exposure resulted in fetal overexposure to maternal GC. However, whether prenatal caffeine ingestion could induce high levels of GC in the IUGR fetus to affect the development of the fetal HPA axis, or what the underlying mechanisms may be, is unknown.

In the present study, we aimed to demonstrate that prenatal caffeine ingestion leads to fetal overexposure to maternal GC. An additional goal was to further observe the functional development of the fetal hippocampus and the HPA axis. This work will be beneficial in elucidating the developmental toxicity of caffeine and in exploring the fetal origin of adult HPA axis dysfunction and metabolic syndrome susceptibility for offspring with IUGR induced by caffeine.

## Results

### Prenatal Caffeine Ingestion Induces IUGR and Inhibits the Development of the Fetal HPA Axis

**Figure 1:**
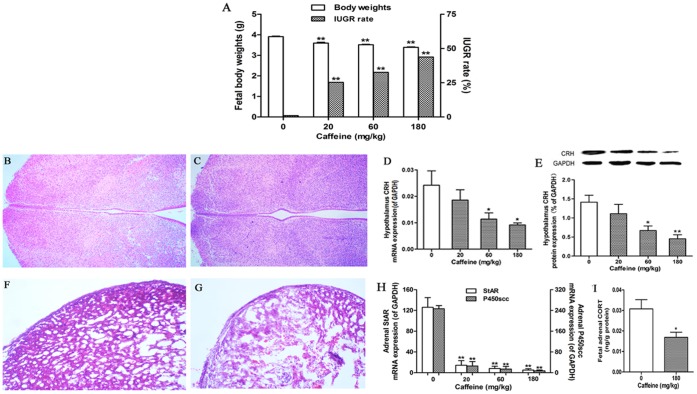
Caffeine-induced fetal growth retardation and inhibition of the fetal hypothalamic–pituitary–adrenal axis. Pregnant rats ingested different doses of caffeine (20, 60, and 180 mg/kg) once per day from gestational day (GD) 11 to GD20. On GD20, the pregnant rats were anesthetized, and all of the fetuses in each group were weighed for calculation of the intrauterine growth retardation (IUGR) rate. One whole fetal brain and one pair of fetal adrenal glands were randomly selected from each group and processed for hematoxylin and eosin (HE) staining. The fetal hypothalamus and adrenal glands were also dissected under an anatomy scope and collected. Fetal tissues from each single pregnant rat were counted as one sample. (A) Fetal body weight and IUGR rate. (B, C) Histopathology of the fetal hypothalamus in the control (B) and caffeine groups (C), magnification 100×. (D, E) Real-time quantitative RT-PCR and Western blotting detection of the mRNA and protein expression of the hypothalamus corticotropin-releasing hormone (CRH). (F, G) Histopathology changes in the fetal adrenal glands in the control (F) and caffeine groups (G), magnification 200×. (H) Real-time quantitative RT-PCR detection of the mRNA expression of the adrenal steroid acute regulatory protein (StAR) and cholesterol side-chain cleavage enzyme (P450scc). (I) ELISA detection of the endogenous level of fetal adrenal corticosterone (CORT). The CORT content was expressed relative to the adrenal protein measured using the BCA protein detection kit. For Western blotting detection, the bar graphs represent the quantitative densitometric results of CRH protein expression, the intensity of the band of each sample was normalized on the basis of GAPDH protein content. For real-time quantitative RT-PCR detection, each sample was normalized on the basis of GAPDH mRNA content. Mean ± SEM, n = 8 pregnant rats. ^*^ *p*<0.05, ^**^ *p*<0.01 vs. control.

As shown in Fig. 1A, prenatal caffeine (20, 60, and 180 mg/kg·d) ingestion significantly decreased fetal body weight to 86.7–92.1% of the control (*p*<0.01) and increased the IUGR rate to 25.3–43.8% (*p*<0.01) at gestational day (GD) 20.

No remarkable pathological changes in the fetal hypothalamus were observed between the control (Fig. 1B) and the caffeine groups (180 mg/kg·d, Fig. 1C); however, caffeine treatment resulted in a significant decrease in mRNA and protein expression of fetal hypothalamus corticotrophin-releasing hormone (CRH) (*p*<0.05 or *p*<0.01, Figs. 1D and 1E). Compared with the control (Fig. 1F), the fetal adrenal cortex changed into slight after caffeine treatment (180 mg/kg·d), presenting a markedly reduced cell number and a disorganized cell arrangement (Fig. 1G). Furthermore, caffeine treatment significantly decreased the mRNA expression of fetal adrenal StAR and P450scc (*p*<0.01, Fig. 1H), as well as the level of fetal adrenal endogenous corticosterone (CORT) (*p*<0.05, Fig. 1I).

### Prenatal Caffeine Ingestion Induces Fetal Overexposure to Maternal GC and Activated GC Metabolism in the Fetal Hippocampus

**Figure 2:**
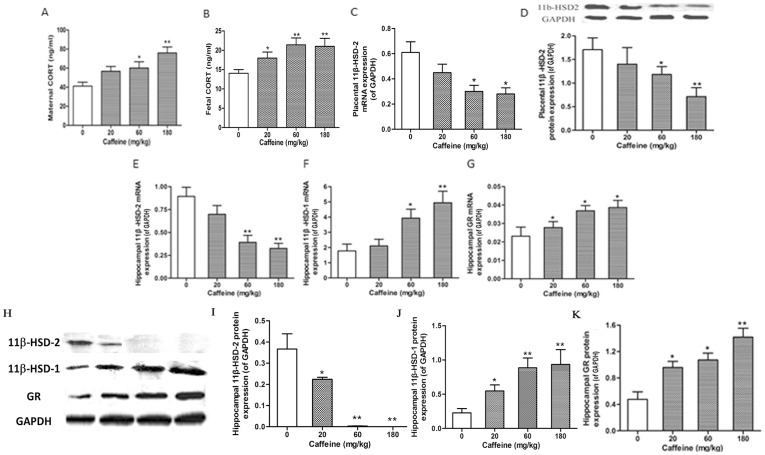
Caffeine-induced fetal overexposure to maternal glucocorticoid and glucocorticoid metabolic activation in the fetal hippocampus. Pregnant rats ingested different doses of caffeine (20, 60, and 180 mg/kg) once per day from gestational day (GD) 11 to GD20. On GD20, the pregnant rats were anesthetized and sacrificed. The placenta and fetal serum prepared from blood samples were collected. The fetal hippocampuses were also dissected under an anatomy scope and collected. Placenta, fetal blood, and fetal hippocampus from each single pregnant rat were counted as one sample. (A, B) ELISA detection of the levels of maternal and fetal blood corticosterone (CORT). (C, D) Real-time quantitative RT-PCR and Western blotting detection of the mRNA and protein expression of the placental 11β-hydroxysteroid dehydrogenase 2 (11β-HSD-2). (E–K) Real-time quantitative RT-PCR and Western blotting detection of the mRNA and protein expression of the hippocampal 11β-HSD-2, 11β-hydroxysteroid dehydrogenase 1 (11β-HSD-1), and glucocorticoid receptor (GR). For real-time quantitative RT-PCR detection, each sample was normalized on the basis of GAPDH mRNA content. For Western blotting detection, the bar graphs represent the quantitative densitometric results of 11β-HSD-2, 11β-HSD-1 or GR protein expression; the intensity of each band of each sample was normalized on the basis of GAPDH protein content. Mean ± SEM, n = 8 pregnant rats. ^*^ *p*<0.05, ^**^ *p*<0.01 vs. control.

In the caffeine-treated groups, the levels of maternal and fetal blood CORT were higher, whereas mRNA and protein expression of placental 11β-HSD-2 were lower relative to the control group. These responses tended to be dose-dependent (*p*<0.05 or *p*<0.01, Figs. 2A–2D). Further, mRNA and protein expression of 11β-HSD-2 were lower, while mRNA and protein expression of 11β-hydroxysteroid dehydrogenase 1 (11β-HSD-1), as well as glucocorticoid receptor (GR) in the fetal hippocampus in the caffeine-treated groups, were higher than those in the control group (*p*<0.05, Figs. 2E–2K).

### Direct Effect of Caffeine on GC Metabolic Activation in the Fetal Hippocampus

**Figure 3:**
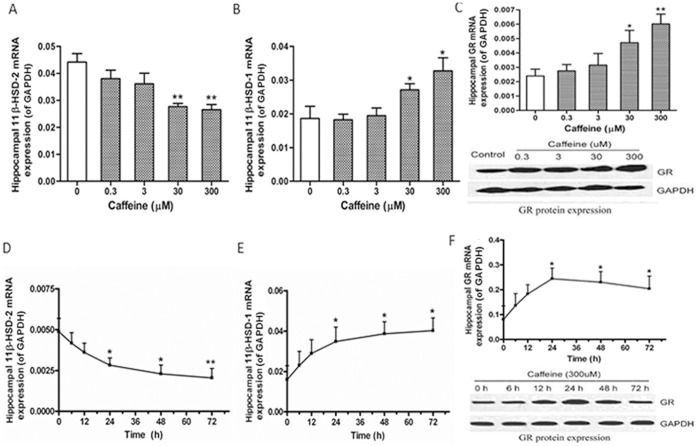
Caffeine-induced glucocorticoid metabolic activation in fetal hippocampal neurons *in vitro*. Primary fetal hippocampal neurons isolated from fetal brains on gestational day 20 were cultured with 300 µM caffeine for 0–72 h or with several different concentrations of caffeine (0, 0.3, 3, 30, and 300 µM) for 24 h. After treatment, the cells were collected for analysis. (A–C) Real-time quantitative RT-PCR and/or Western blotting detection of the mRNA and/or protein expression of 11β-hydroxysteroid dehydrogenase 2 (11β-HSD-2), 11β-hydroxysteroid dehydrogenase 1 (11β-HSD-1), and glucocorticoid receptor (GR) after 0 to 300 µM caffeine treatment for 24 h. (D–F) Real-time quantitative RT-PCR and/or Western blotting detection of the mRNA and/or protein expression of 11β-HSD-2, 11β-HSD-1, and GR after 300 µM caffeine treatment for 0 to 72 h. For real-time quantitative RT-PCR detection (n = 5), each sample was normalized on the basis of GAPDH mRNA content. Data are expressed as the mean ± SEM, ^*^ *p<*0.05, ^**^ *p<*0.01 vs. control. For Western blotting detection (n = 3), representative results were presented for demonstrating the protein expressions of GR.

We also examined the expression of 11β-HSD-2, 11β-HSD-1, and GR in the caffeine-treated primary fetal rat hippocampal neuron *in vitro*. No cytotoxic effect was observed up to 300 µM of caffeine treatment for 72 h (MTT data not shown). The dose–effect relationship data show that 0 to 300 µM caffeine dose-dependently reduced the 11β-HSD-2 mRNA expression but increased the mRNA and/or protein expression of 11β-HSD-1 and GR within 24 h (*p*<0.05 or *p*<0.01, Fig. 3A–3C). The time–effect relationship data also reveal that 300 µM caffeine reduced the 11β-HSD-2 mRNA expression in a time-dependent manner within 72 h but increased the mRNA and/or protein expression of 11β-HSD-1 and GR and that GR expression reaches a peak at 24 h (*p*<0.05 or *p*<0.01, Figs. 3D–3F).

### Direct Effect of Caffeine on the Hypermethylation of the 11β-HSD-2 Promoter in the Fetal Hippocampus

The promoter methylation state of 15, 398 genes of the primary fetal rat hippocampal neuron after 24 h of treatment with 300 µM caffeine was examined using the RN34 RefSeq promoter methylation array provided by Roche NimbleGen Inc. (Wisconsin, USA). The results show that the promoter methylation state of 1679 genes were changed in the caffeine group; of these genes, 1183 were hypermethylated and 496 genes were hypomethylated compared with the control. Hypermethylation was observed in nt −358 to −77 of the 11β-HSD-2 (Gene ID: 25117) promoter in the caffeine group, where the methylation peak score was 2.51. However, no significant methylation change was found in the promoters of the 11β-HSD-1 and GR genes.

**Figure 4:**
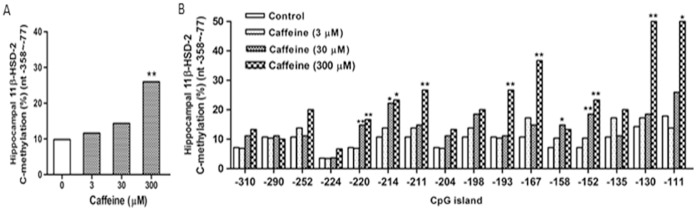
Caffeine-induced hypermethylation of 11β-hydroxysteroid dehydrogenase 2 (11β-HSD-2) promoter in fetal hippocampal neurons *in vitro*. The primary fetal hippocampal neurons isolated from fetal brains on gestational day 20 were cultured with several different concentrations of caffeine (0, 3, 30, and 300 µM) for 24 h. After treatment, the methylation status of 11β-HSD-2 gene was detected by bisulfite-sequencing PCR method. (A, B) Ratio of the total methylation and each CpG site methylation in the 11β-HSD-2 promoter region (nt −358 to −77) (n = 5). ^*^ *p<*0.05, ^**^ *p<*0.01 vs. control.

Gene profiling analysis indicates that there are 16 CpGs within nt −358 to −77 of the rat 11β-HSD-2 promoter. Based on the bisulfate sequencing PCR (BSP) results, caffeine was shown to dose-dependently increase the total methylation ratio within nt −358 to −77 of the 11β-HSD-2 promoter, most notably in the group treated with 300 µM caffeine for 24 h, resulting in 27.3% of the total methylation ratio, which is higher than 9% of the control (*p*<0.01, Fig. 4A). In addition, caffeine also significantly increased the methylation ratio of each single CpG site; that is, 30 µM of caffeine increased the methylation at nt −220, −214, −158, and −152, and 300 µM of caffeine increased the methylation at nt −220, −214, −211, −193, −167, −152, −130, and −111 (*p*<0.05 or *p*<0.01, Fig. 4B).

## Discussion

The HPA axis is an essential neuroendocrine axis that includes the hypothalamic CRH, pituitary ACTH, adrenal GC, and their corresponding receptors. The hypothalamus serves as a “transfer station” to receive signals from the superior regulation center and regulate the release of pituitary ACTH and adrenal GC. The adrenal, as the terminal effector organ of the HPA axis, secretes GC and plays a crucial role in individual growth and development [27]. Cholesterol, as the substrate for GC synthesis, needs to be transferred from the outer mitochondrial membrane to the inner membrane, where P450scc cleaves the cholesterol side chain to produce pregnenolone, and StAR is the transport protein that regulates cholesterol transfer within the mitochondria. Accordingly, both StAR and P450scc are essential for GC synthesis [28]. The results of this study indicate that the expression of fetal hypothalamic CRH was remarkably reduced, the fetal adrenal cortex changed into slight and the expression of StAR and P450scc, as well as the level of fetal adrenal endogenous CORT, were all significantly decreased after caffeine treatment, suggesting that prenatal caffeine ingestion inhibits the functional development of the fetal HPA axis.

In physiological conditions, the placental barrier builds and maintains a GC concentration gradient (approximately 10-fold) between the mother and the fetus during pregnancy [29]. The placental 11β-HSD-2 is a GC-inactivated enzyme responsible for preventing the fetus from being overexposed to the maternal GC [30]. Hence, placental 11β-HSD-2 is a crucial factor in determining the fetal GC level [31]. In the present study, we found that the level of blood CORT in the mother was increased after caffeine treatment, which was consistent with our recent report that caffeine increased cortisol production in human adrenocortical cells [21]. Meanwhile, we found that the expression of placental 11β-HSD-2 was reduced, and the level of CORT in fetal blood was increased after caffeine treatment. Thus, we proposed that prenatal caffeine ingestion-induced high levels of GC in the fetus mainly come from the mother rats and that this high level of GC could inhibit the activity of the fetal HPA axis through a negative feedback regulation. Our recent report also demonstrated that prenatal dexamethasone (a synthetic glucocorticoid) treatment led to obvious HPA axis suppression in IUGR fetal rats [32].

The hippocampus, as the important high negative feedback regulatory center of the HPA axis, is the most sensitive and vulnerable neural target site for circulatory GC [33]. *In utero*, fetal hippocampal GC regulates the functional development of the HPA axis by a negative feedback loop, which is mediated via predominantly expressed GR during the time of fetal development [34], [35]. The GR expression-dependent regulation of the HPA axis in the fetal hippocampus is gradually established with the progression of pregnancy and maturation of the central nervous system [36], [37]. Studies by Weaver [38], [39] further suggest a causal relationship between GR expression and the response of the HPA axis in rat offspring during the perinatal period. All of these results indicate a causative link between fetal hippocampal GC/GR and the development of the fetal HPA axis. Hippocampal 11β-HSD is involved in regulating the binding of GC to GR. 11β-HSD can be subdivided into types 1 and 2 that catalyze interconversion of active glucocorticoids (cortisol in humans and corticosterone in rodents) and the inactive 11-keto form (cortisone in humans and 11-dehydrocorticosterone in rodents), thereby modulating the concentration and function of hippocampal GC. 11β-HSD-2 is widely expressed in fetal brain during the mid-embryonic development period, while 11β-HSD-1 and GR are limitedly expressed in fetal brain at mid-embryonic development, reaching their peaks in the late embryonic development period and remaining until after birth [40]–[42]. Research has demonstrated that 11β-HSD-1 and GR are colocalized in the same hippocampal neuron [43]. GC was implicated in the upregulation of 11β-HSD-1 activity via a positive feedback loop regulation of GR, and enhanced 11β-HSD-1 activity may further enhance the local active GC levels in the hippocampus [29], [44], [45]. In this study, 11β-HSD-1 and GR expression increased in the fetal hippocampus after prenatal caffeine ingestion, suggesting that fetal overexposure to maternal GC induced by caffeine could inhibit the activity of the fetal HPA axis via enhanced hippocampal 11β-HSD-1 and GR through a negative feedback regulation.

Low molecular weight lipophilic caffeine readily crosses the placental barrier and reaches the fetus, where it is mainly distributed in the brain and liver [46], [47]. In addition, caffeine is difficult to metabolize and excrete because of its prolonged circulation half-life [46], [48], resulting in a high accumulation in the fetus. Using nuclear magnetic resonance (NMR) spectroscopy, we have detected the existence of caffeine in fetal blood after prenatal caffeine ingestion. In the current study, we further demonstrated that caffeine not only inhibited the 11β-HSD-2 expression but also intensified the expression of 11β-HSD-1 and GR in the primary fetal hippocampal neuron *in vitro*, and such effects showed a favorable dose- and time-dependent manner. Therefore, caffeine was proven to have a direct action on 11β-HSD and GR expression in the fetal hippocampus, which is consistent with the *in vivo* results. The reduced expression of 11β-HSD-2 by caffeine may increase the levels of active GC, as well as the expression of 11β-HSD-1 and GR in the fetal hippocampus, which in turn further inhibit the function and development of the fetal HPA axis.

Epigenetic dysregulation is an important molecular mechanism through which a prenatal insult such as xenobiotic exposure can persistently affect the postnatal phenotype [49], [50]. Mechanisms such as DNA CpG methylation affect gene expression by regulating the transcriptional machinery and transcriptional factor access to DNA. The 11β-HSD-2 promoter contains highly GC-rich regions (80% of the sequence) that have been shown to be epigenetically regulated, both *in vitro* and *in vivo* [51]. Therefore, in the present study, we propose that epigenetic regulation is a good candidate mechanism for hippocampal 11β-HSD-2 expression. For rat 11β-HSD-2, the promoter region next to the transcriptional start site is rich in binding sites for transcriptional enhancers, namely, the specificity protein 1 (Sp1) and NF-kappa B (NF-κB) p65. Basega *et al*. [52] recently reported that in a rat IUGR model induced by a bilateral uterine artery ligation during the embryonic period, a persistent decrease occurred in kidney 11β-HSD-2 mRNA and protein expression. These authors suggested that the modified methylation pattern in the CpG sites of the 11β-HSD-2 promoter, as well as the changes in the corresponding transcriptional factor binding, possibly leads to reduced 11β-HSD-2 expression. In the present study, we demonstrated that caffeine enhanced the methylation ratio of multiple single CpG sites, as well as the total methylation ratio at nt −358 to −77 of the hippocampal 11β-HSD-2 promoter. Furthermore, the methylated sites at nt −214 and −152 act as binding sites for Sp1 and NF-κBp65 [52], indicating that caffeine may inhibit the binding of Sp1 and NFκB p65 to the promoter via methylation of these binding sites, resulting in the inhibition of 11β-HSD-2 expression.

**Figure 5:**
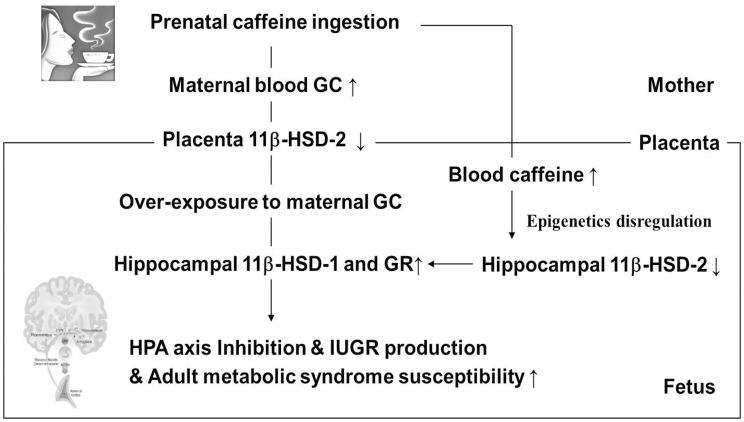
Caffeine-induced intrauterine hypothalamic–pituitary–adrenal (HPA) axis-related neuroendocrine alteration in intrauterine growth retardation (IUGR) rats. GC, glucocorticoid; 11β-HSD-2, 11β-hydroxysteroid dehydrogenase 2; 11β-HSD-1, 11β-hydroxysteroid dehydrogenase 1; GR, glucocorticoid receptor.

In summary, our present findings suggest that prenatal caffeine ingestion induces an intrauterine HPA axis-related neuroendocrine alteration in the IUGR rat model (Fig. 5). In brief, prenatal caffeine ingestion can elevate the maternal GC level and inhibit the placental 11β-HSD-2 expression, thereby causing a fetal overexposure to maternal GC. Moreover, caffeine can enter the fetus and directly inhibit the fetal hippocampal 11β-HSD-2 expression by epigenetic dysregulation. These changes subsequently enhanced the expression of 11β-HSD-1 and GR in the fetal hippocampus, which inhibit the fetal HPA axis and retard the fetal development. Additional results from our group further revealed that using nuclear magnetic resonance (NMR)-based metabonomics technology, we observed that prenatal caffeine ingestion could characteristically change the fetal metabonome, which is most likely attributed to the alterations in glucose and lipid metabolic pathways induced by increased circulatory GC level [53]. In addition, in our recent parallel experiment, caffeine-induced alterations in the fetal HPA axis and in glucose and lipid metabolism could be maintained until the postnatal period and were found to enhance the susceptibility to adult metabolic syndrome and fatty liver (unpublished data). Therefore, these results will be beneficial in elucidating the fetal origin of adult HPA axis dysfunction and metabolic syndrome susceptibility for offspring with IUGR induced by caffeine.

According to a survey, the average caffeine intake in pregnant women from western countries is approximately 144 mg/d (2.06 mg/kg·d) [54]. Using the dose conversion between humans and rats (human:rats1∶6.17) [55], the lowest dose of caffeine ingestion (20 mg/kg·d) by pregnant rats in the present study is 1.5-fold higher than the reported data, which is equivalent to 1.5–2.2 cups of daily coffee intake by pregnant women (according to a previous investigation [56], a standard cup of coffee contains 100–150 mg of caffeine on average). Although the fetal body weight is approximately 90% that of the control, a series of important indices reflecting fetal growth and development (such as StAR and P450scc in fetal adrenal glands) were significantly decreased to 10%–13% of those in the control under a lower caffeine treatment dose, suggesting that daily caffeine ingestion during pregnancy can inhibit the fetal HPA axis development. Therefore, caffeine ingestion during pregnancy should be avoided.

## Materials and Methods

### Chemicals and Reagents

Caffeine (CAS No. C0750), polylysine, and 0.25% EDTA-trypsin were purchased from Sigma-Aldrich Co., Ltd. (St Louis, MO, USA). Rat CORT ELISA kits was obtained from R&D Systems, Inc. (Minneapolis, MN, USA). RNA-Solv Reagent and HiBindTM PCR DNA extraction kit were provided by Omega Bio-Tek Inc. (Norcross, GA, USA). The reverse transcription and real-time RT-PCR kits were purchased from Takara Biotechnology Co., Ltd. (Dalian, China). The protein detection kit was from Bio-Rad Laboratories, Inc. (Hercules, CA, USA). The enhanced chemiluminescence kit (ECL) and goat anti-rabbit IgG were obtained from Pierce Biotechnology Inc. (Rockford, IL, USA). The genome DNA isolation kit (DNeasy Blood & Tissue Kit) was provided by Qiagen Co., Ltd. (Dusseldorf, Germany). The EZ DNA Methylation-direct kit was from Zymo Research Co., Ltd. (Irvine, CA, USA). The Platinum Taq PCRx DNA Polymerase kit, Dulbecco's modified Eagle's medium (DMEM), fetal bovine serum (FBS), neurobasal medium, and B27 nerve growth factor were purchased from Invitrogen Co., Ltd. (Carlsbad, CA, USA). The pGEM-T Easy vector was purchased from Promega Co. (Madison, WI, USA). l-glutamine was imported from the USA and repackaged by Shanghai Sino-American Biotechnology Co., Ltd. (Shanghai, China). The polyclonal antibodies against rat 11β-HSD-2, 11β-HSD-1, GR, and CRH were purchased from Santa Cruz Biotechnology Inc. (Santa Cruz, CA, USA). The oligonucleotide primers of rat 11β-HSD-2 and others were synthesized by Sangon Biotech Co., Ltd. (Shanghai, China). Isoflurane was purchased from Baxter Healthcare Co. (Deerfield, IL, USA). The other chemicals and reagents were of analytical grade.

### Animals and Treatment

Specific pathogen-free Wistar rats weighing 200 g ±20 g (female) or 280 g ±20 g (male) were obtained from the Experimental Center of Hubei Medical Scientific Academy (No. 2006–0005, Hubei, China). The animals were allowed to acclimate for at least one week before being subjected to experimental conditions.

For mating, two female rats were placed together with one male rat overnight. The day at which the evidence of mating (i.e., vaginal plug or vaginal smear with sperm cells) was observed was designated as GD0. The animals were housed under standard conditions and allowed free access to standard food and water. From GD11 to GD20, the pregnant rats ingested different doses of caffeine (20, 60, and 180 mg/kg) once per day, and the control group received the same volume of the vehicle. On GD20, the pregnant rats were anesthetized with isoflurane and then sacrificed. Each feto-placental unit was quickly removed from the uterus, and the fetuses were weighed after being dried on filter papers. The number of pregnant rats in each group was set to 8 (the litter size of each pregnant rat was 8 to 14 at birth). All of the fetal rats in each group were taken into account to calculate the IUGR rate, according to previously reported criteria for IUGR evaluation (IUGR was diagnosed when the body weight of each individual animal from the treated group was two standard deviations less than the mean body weight of the control group) [1]. One whole fetal brain and one pair of fetal adrenal glands were randomly selected from the control and the caffeine groups (180 mg/kg·d) for fixing in a 10% formaldehyde solution, embedding in paraffin and processing for light microscopy. Meanwhile, a series of fetal tissues, including hippocampus, hypothalamus and adrenal glands, was dissected under an anatomy scope and collected. The maternal and fetal serum prepared from blood samples, as well as the placenta, were also collected. All samples were immediately frozen in liquid nitrogen and then stored at −80°C for the subsequent experiments.

The animal work in this study was performed at the Center for Animal Experiment of Wuhan University (Wuhan, Hubei, P.R. China), which has been accredited by the Association for Assessment and Accreditation of Laboratory Animal Care International (AAALAC International). All animal experiment procedures were approved by and performed in accordance with the Guidelines for the Care and Use of Laboratory Animals of the Chinese Animal Welfare Committee.

### Histopathological Examination

Fetal brain and adrenal specimens were fixed overnight in a 10% formaldehyde solution and processed via the paraffin slice technique. Sections approximately 5 µm thick were stained with hematoxylin and eosin (HE). The morphology of the fetal hypothalamus, as well as the fetal adrenals, was observed under a light microscope.

**Table 1:** Oligonucleotide primers and PCR conditions of rat in quantitative real-time PCR.

Genes	Forward primer	Reverse primer	Product (bp)	Annealing
11β-HSD-1	GGAGCCCATGTGGTATTGA	AGTGCCGGCAATGTAGTGA	105	61°C, 20 s
11β-HSD-2	AGGGGACGTATTGTGACCG	GCTGGATGATGCTGACCTTG	154	63°C, 20 s
GR	CACCCATGATCCTGTCAGTG	AAAGCCTCCCTCTGCTAACC	156	60°C, 20 s
CRH	TGGATCTCACCTTCCACCTTCTG	CCGATAATCTCCATCAGTTTCCTG	103	62°C, 20 s
StAR	GGGAGATGCCTGAGCAAAGC	GCTGGCGAACTCTATCTGGGT	188	65°C, 20 s
P450scc	GCTGCCTGGGATGTGATTTTC	GATGTTGGCCTGGATGTTCTTG	156	63°C, 20 s
GAPDH	TAAAGAACAGGCTCTTAGCACA	AGTCTTGGAAATGGATTGTCTC	107	59°C, 15 s

11β-HSD-1, 11β-hydroxysteroid dehydrogenase 1; 11β-HSD-2, 11β-hydroxysteroid dehydrogenase 2; GR, glucocorticoid receptor; CRH, corticotropin releasing hormone; StAR, steroid acute regulatory protein; P450scc, cholesterol side chain cleavage enzyme; GAPDH, glyceraldehyde phosphate dehydrogenase.

### Blood CORT Detection

Ten microliters of serum were used for the detection of CORT concentrations using an ELISA kit following the manufacturer's protocol.

### Endogenous CORT Detection

Fetal adrenal glands were homogenized with 20% ethanol in phosphate buffer solution (PBS), then centrifuged at 1200×g at 4°C for 5 min. Supernatants were collected for CORT analysis, and the pellets were resuspended in 1 N NaOH for the measurement of protein content. Adrenal CORT content was measured by an ELISA kit, following the manufacturer's protocol. CORT content was expressed relative to adrenal protein measured using the BCA protein detection kit.

### Primary Culture of the Hippocampal Neuron Isolated from Fetal Rats

The pregnant rats were anesthetized with isoflurane and sacrificed on GD20, and the fetuses were rapidly removed. Fetal brains were collected and placed on ice. The hippocampuses from the collected fetal brains were dissected and isolated under an anatomy scope in an ice-cold buffer consisting of 136 mM NaCl, 5.4 mM KCl, 0.2 mM Na_2_HPO_4_, 2 mM KH_2_PO_4_, 16.7 mM glucose, 20.8 mM saccharose, 0.0012% phenol red, and 10 mM HEPES (pH 7.4). The isolated hippocampuses were mechanically triturated and then digested in a solution containing 0.25% trypsin and 1 mM EDTA at 37°C for 10 min. A single cell suspension was obtained by repeated passages of the dissociated tissues through a flame-polished pipette in a plating medium (DMEM supplemented with 10% heat-inactivated FBS). The cells were finally plated on poly-l-lysine-coated dishes (0.1 mg/ml) at optimal cell densities. The cultures were kept at 37°C in a humidified 5% CO_2_-containing atmosphere. The day of dissection was assigned as day 0 (D0). On D1, the serum-containing plating medium was replaced with a growth medium (serum-free neurobasal medium supplemented with 2% B27 supplement and 0.4% glutamine). On D4, D7, and D10, half of the growth medium was changed. On D10, the cells were treated with 300 µM caffeine for 0–72 h or with several different concentrations of caffeine (0, 0.3, 3, 30, and 300 µM) for 24 h. After treatment, the cells were collected for further analysis. The caffeine concentrations (0.3–300 µM) in the present *in vivo* study were designed according to a previous report that 105–310 µM caffeine corresponds to concentrations transferred by the placenta of heavy caffeine consumers [57].

### Real-time Quantitative RT-PCR and Western Blotting

Total RNA and protein were isolated from the fetal tissues (hippocampus, hypothalamus, and adrenal glands), placentas, and cultured hippocampal neurons using the Trizol reagent, following the manufacturer’s protocol. The same tissues of each littermate were pooled for homogenization. After DNAse I (RNase-free) treatment, the concentration and purity of the isolated RNA were determined using a spectrophotometer and adjusted to 1 µg/µl. Total RNA was stored in diethyl pyrocarbonate-H_2_O (DEPC-H_2_O) at −80°C. The protein concentration was measured using a Bio-Rad protein assay kit.

For real-time quantitative (RT-PCR) analysis, single-strand cDNA was prepared from 2 µg of total RNA according to the protocol of the Exscript RT reagent kit. Primers were designed using Primer Premier 5.0, and their sequences are shown in Table 1. Relative standard curves were constructed for the following target rat genes: GR, CRH, StAR, P450scc, 11β-HSD-1, and 11β-HSD-2, as well as the housekeeping gene glyceraldehyde phosphate dehydrogenase (GAPDH), using the corresponding RT-PCR products isolated by DNA extraction kit with different concentrations ranging from 10^−3^–10^−9 ^pg per reaction. PCR assays were performed in 36-well optical reaction plates using the RG-3000 Rotor-Gene 4 Channel Multiplexing System (Corbett Research Pty Ltd., Sydney, Australia) at a total volume of 25 µl reaction mixture containing 2 µl of 0.1 µg/µl cDNA template, 0.5 µl of 10 µmol/l of each primer, 12.5 µl of 2× Premix Ex Taq, 0.5 µl of 20× SYBR Green I, and 9 µl of DEPC-H_2_O. To quantify the transcripts of the genes precisely, the expression of GAPDH mRNA was used as the quantitative control, and each sample was normalized on the basis of GAPDH mRNA content. The PCR cycling conditions were as follows: 95°C, 10 s for pre-denaturation; 95°C, 5 s for denaturation; appropriate annealing conditions for each gene (listed in Table 1); 72°C, 15 s for elongation (this step only for the GAPDH reaction). The primer sequence of GR is according to the previous reports [58]–[61]. We identified a point mutation in the sense primer and synthesized a new one with correct sequence. We also performed some experiments to make sure that such point mutation may not alter much of the actual result (Supporting Information S1).

For the Western blotting analysis, 50 µg of protein per well was loaded on a 12% sodium dodecyl sulfate–polyacrylamide electrophoresis (SDS/PAGE) gel and resolved via standard electrophoresis. The gels were then electrophoretically transferred onto nitrocellulose membranes. The membranes were blocked with 5% bovine serum albumin (BSA) in PBS containing 0.1% Tween 20 (PBS-T) for 1 h at room temperature and then probed overnight at 4°C using polyclonal antibodies against 11β-HSD-2, 11β-HSD-1, GR, and CRH at a final dilution of 1∶400 (w/v). Protein molecular weight markers were included on each blot. To verify the loading of same amount of samples, the membranes were probed overnight at 4°C using polyclonal GAPDH antibodies at a final dilution of 1∶600 (w/v). The membranes were extensively washed in PBS with Tween-20 and incubated with peroxidase-conjugated anti-rabbit IgG (1∶3000 w/v). The bands were visualized using an ECL kit, and their optical densities were measured using the Photo Documentation and Imaging System (BIO-ID VL, Conn France).

### Methylation Array and BSP

An RN34 RefSeq promoter methylation array (No. C4226-00-01, Roche NimbleGen Inc., Madison, WI, USA) was used to detect the changes in the promoter methylation state of 15,398 genes in the cultured hippocampus neuron after 300 µM caffeine treatment for 24 h. The extraction of sample genomic DNA, DNA quantification, immunoprecipitation and hybridization of the methylated DNA, chemiluminescence detection, image acquisition, and data analysis were all conducted by Kang Cheng Biotechnology Co., Ltd. (Shanghai, China). All these procedures were in compliance with the MIAME Guidelines.

For the BSP assay, genomic DNA from the cultured hippocampus neuron was isolated using the genome DNA isolation kit and 1 µg of DNA for sodium bisulfite modification, which was performed using the EZ DNA Methylation kit according to the manufacturer's guidelines. In brief, the sodium bisulfite modification chemically converted the non-methylated cytosines to uracils, whereas the methylated cytosines were protected from this conversion.

The 11β-HSD-2 promoter (nt −358 to −77) was amplified using primers (forward: GTAGTGGTGTGTGAGGAAGTATG; reverse: AAAACTTTCCTCCACTTCTATCTCA). The PCR mixtures contained 50 ng modified DNA, 0.5 µM primers, 200 µM dNTPs, 1.5 mM MgCl_2_, and 1 U Platinum Taq DNA Polymerase. The amplification conditions were as follows: denaturation at 95°C for 2 min; 40 cycles of 95°C for 30 s, 60°C for 30 s, and 72°C for 30 s; a final elongation step of 72°C for 10 min. Subsequently, the PCR products were purified using a Qiagen PCR purification kit and cloned into a pGEM-T vector. Ten cloned PCR fragments for every one sample were sequenced by Sunbiotech Co., Ltd. (Beijing, China).

### Statistics

Excel (Microsoft, Redmond, WA, USA) and Prism (GraphPad Software, La Jolla, CA, USA) were used for data analysis. All presented measurement data are expressed as the mean ± SEM and were evaluated with one-way ANOVA followed by a post hoc Dunnett–t-test. A Chi-square analysis was performed to test for a difference in the proportions of the categorical variables between groups, such as the IUGR rate and the methylation rate. Statistical significance was set at *p*<0.05.

## Supporting Information

Supporting Information S1 **The melt curve of glucocorticoid receptor (GR) and the image of complete agarose gel of GR band at 156 bp using two sense primers.** Sense Primer 1∶5′-CACCCATGATCCTGTCAGTG-3′; Sense Primer 2∶5′- CACCCATGACCCTGTCAGTG-3′.
(DOCX)
