# Supplementary material for: Caffeine-Induced Activated Glucocorticoid Metabolism in the Hippocampus Causes Hypothalamic-Pituitary-Adrenal Axis Inhibition in Fetal Rats
Source: PLoS One. 2012 Sep 6;7(9):e44497. doi: 10.1371/journal.pone.0044497 (PMC3435254; doi:10.1371/journal.pone.0044497)
Supplement: Supporting Information S1 — The melt curve of glucocorticoid receptor (GR) and the image of complete agarose gel of GR band at 156 bp using two sense primers. Sense Primer 1∶5′-CACCCATGATCCTGTCAGTG-3′; Sense Primer 2∶5′- CACCCATGACCCTGTCAGTG-3′. (DOCX) [file pone.0044497.s001.docx]

We identified a point mutation in the glucocorticoid receptor (GR) sense primer (Sense Primer 1: 5’-CACCCATGATCCTGTCAGTG-3’). We have synthesized a new one with correct sequence (Sense Primer 2: 5’- CACCCATGACCCTGTCAGTG-3’) and performed an apple-to-apple real time RT-PCR experiment to compare the two sense primers. The result indicated a single peak of melt curve of GR using two sense primers. Further, we performed a PCR experiment without RT reaction on total RNA that were pre-treated with RNase-free DNase (Takara Biotechnology Co., Ltd), and we got an image of complete agarose gel, in which a single band at 156 bp was present with both sense primers. These results suggest that such point mutation may not alter much of the actual result.


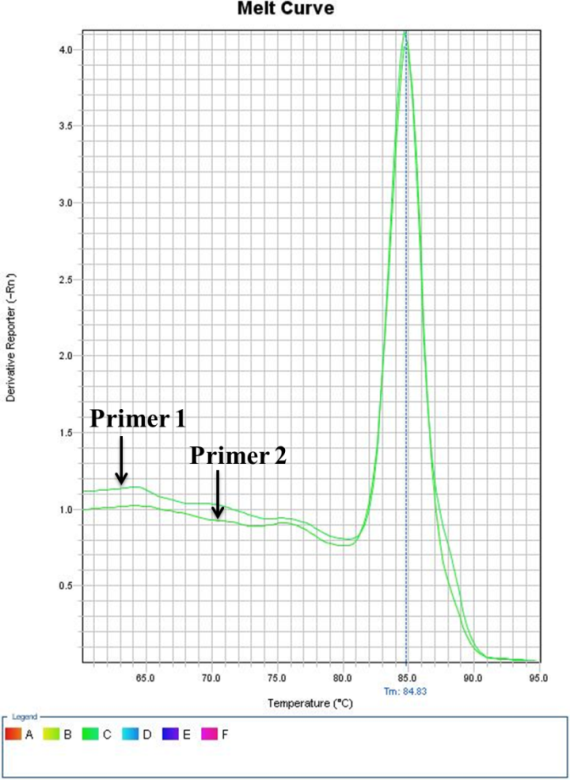

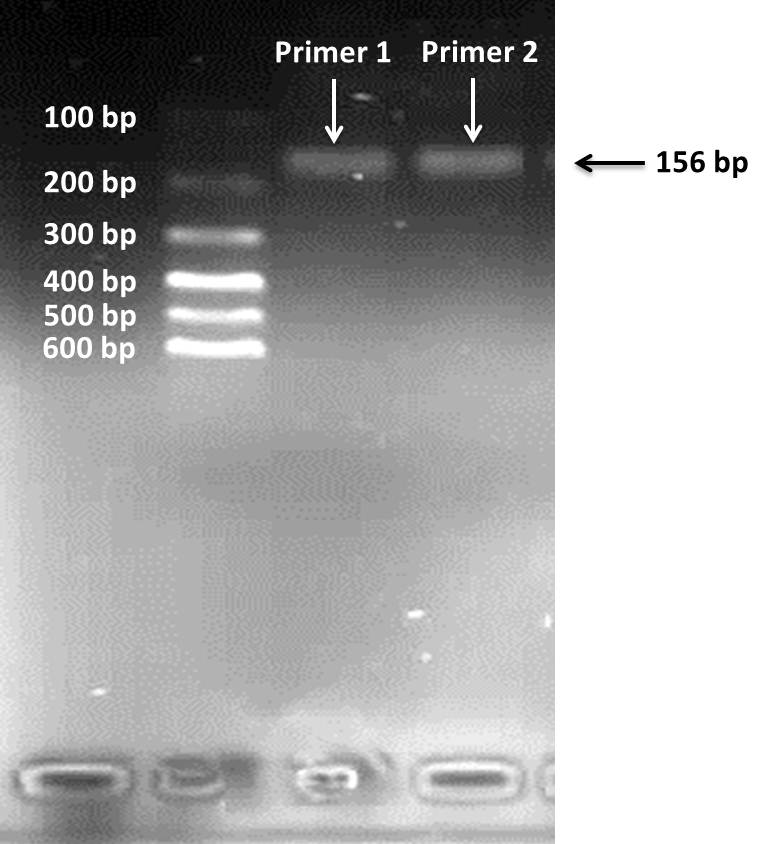


The melt curve of glucocorticoid receptor (GR) and the image of complete agarose gel of GR band at 156 bp using two sense primers. Sense Primer 1: 5’-CACCCATGATCCTGTCAGTG-3’; Sense Primer 2: 5’- CACCCATGACCCTGTCAGTG-3’
